# Clinical efficacy of STMIPO in the management of distal one-third fibular fractures

**DOI:** 10.3389/fsurg.2026.1848647

**Published:** 2026-07-06

**Authors:** Wei Li, Yang Li, Quankui Zhuang, Song Shao

**Affiliations:** 1 Department of Orthopedics, Lu'an Hospital of Anhui Medical University, Lu'an, China; 2 Department of Orthopedic, No 2 People's Hospital of Fuyang City, Fuyang, Anhui, China

**Keywords:** closed reduction and internal fixation, fibular fracture, MIPO, open reduction and internal fixation, STMIPO

## Abstract

**Background:**

Second to minimally invasive plates osteosynthesis (STMIPO) is a novel surgical technique that combines the small incision, minimally invasive, and aesthetic advantages of minimally invasive plate osteosynthesis (MIPO) with the direct visualization fracture reduction of open reduction and internal fixation (ORIF). This study primarily evaluates the clinical efficacy of STMIPO in the treatment of distal one-third fibular fractures.

**Methods:**

A retrospective analysis was conducted on 145 fibular fracture patients treated in our orthopedic department from January 2021 to December 2023. All surgeries were performed by two main surgeons; one used open reduction and internal fixation (ORIF group), and the other used STMIPO technique (STMIPO group). The study compared the ORIF and STMIPO groups in terms of operation time, fracture healing time, intraoperative blood loss, length of incision, postoperative ankle AOFAS score for isolated fibular fractures, and postoperative complications (such as wound infection, plate exposure, delayed bone healing, and hardware-related pain).

**Results:**

The average follow-up time was 17.12 ± 2.66 months (weighted average of the two groups), during which all patients achieved osseous union of the fibula. Statistical analysis showed that the STMIPO group had advantages over the ORIF group in terms of surgical incision length, intraoperative blood loss, and postoperative complications. There was no significant difference between the two groups in postoperative ankle AOFAS scores for isolated fibular fractures.

**Conclusion:**

STMIPO technique, as a new minimally invasive fracture treatment technique, has demonstrated significant advantages in the treatment of distal one-third fibular fractures, including smaller surgical incisions, minimal damage to surrounding tissues, faster postoperative recovery, and comparable fracture healing.

**Level of evidence:**

Therapeutic Level III.

## Introduction

With the intensification of societal aging, the number of patients with ankle fractures has increased (1). Studies indicate that ankle fractures account for approximately 10% of all fractures (2, 3). The anatomical reduction of the fibula post-fibular fracture surgery plays a crucial role in the stability of the ankle joint (4–7). However, there is often a conflict between achieving anatomical reduction and minimally invasive treatment. Achieving better reduction typically requires more significant trauma, but the greater the trauma, the more it can affect fracture healing.

Currently, the conventional treatment for fibular fractures remains open reduction and internal fixation (8). Common surgical approaches include the lateral approach and the posterolateral approach (9, 10). Internal fixation devices commonly used include locking plates, non-locking plates, and intramedullary nails (11–13).

STMIPO is a minimally invasive treatment technique for limb fractures first proposed by our team. The name ’Second to MIPO’ reflects a theoretical consideration: compared with pure MIPO, STMIPO causes slightly more periosteal injury at the fracture site because it requires a limited open approach to achieve direct visualization of the fracture ends. It is therefore named ’second to’ MIPO in terms of periosteal preservation. However, this minor trade-off enables two critical advantages: anatomical reduction under direct vision—which is difficult to achieve with pure MIPO—and percutaneous plate and screw insertion—which avoids the extensive soft tissue dissection of traditional ORIF. By combining these elements, STMIPO aims to achieve precise fracture reduction and stable internal fixation through the smallest possible incision, balancing the competing goals of reduction quality and soft tissue preservation (14)..

While STMIPO shares technical similarities with previously described ‘hybrid MIPO’ techniques, it possesses five distinctive features inherent to its design. First, its nomenclature — 'Second to MIPO' — honestly acknowledges a theoretical disadvantage (slightly more periosteal injury than pure MIPO), reflecting an act of academic transparency. Second, the exposure is deliberately limited: only a small window (3–4 cm) is created, preserving approximately three-fourths of the periosteal circumference — reduction is achieved through a 'partial view of the whole.' Third, the plate insertion pathway differs from that of MIPO: the plate is inserted from the fracture site outward, allowing precise guidance under direct visualization. Fourth, the technique provides a clear damage hierarchy (MIPO < STMIPO < ORIF) to aid intraoperative decision-making. Fifth, unlike pure MIPO, STMIPO can be readily converted to limited open reduction if difficulties arise — a 'safety net' that has been clinically validated in our practice.

While our previous work confirmed the feasibility of the STMIPO technique across various limb fractures in a small proof-of-concept study, a dedicated comparative evaluation of STMIPO versus ORIF specifically for distal one-third fibular fractures has not yet been performed. The present investigation builds directly upon that work by focusing exclusively on distal one-third fibular fractures. The present study was therefore designed to compare the clinical outcomes of STMIPO versus ORIF in the treatment of distal one-third fibular fractures. Unlike our prior feasibility study, the current study provides a large-sample (*n* = 145) comparison focusing exclusively on distal one-third fibular fractures—an anatomically and functionally distinct fracture type.

## Materials and methods

This study retrospectively analyzed 145 patients with fibular fractures treated with plate fixation surgery in our hospital from January 2021 to December 2023. All surgeries were performed by two main surgeons, with one using STMIPO technique (STMIPO group) and the other using open reduction and internal fixation technique (ORIF group).Patient allocation was determined by the day of hospital admission and the corresponding on-call surgeon's operating schedule, without randomization. The two participating surgeons performed their respective techniques (STMIPO or ORIF) on fixed weekly operating days. Thus, assignment was based on temporal availability rather than patient-specific factors.

In the STMIPO group, there were 74 patients with fibular fractures, including 40 males and 34 females; 13 had isolated fibular fractures, and 61 had tibiofibular fractures. According to the Danis-Weber classification, there were 5 cases of type A fractures, 39 cases of type B fractures, and 30 cases of type C fractures. The average age of the patients was 47.89 ± 17.26 years, with an average follow-up time of 16.85 ± 1.62 months.

In the ORIF group, there were 71 patients with fibular fractures, including 39 males and 32 females; 11 had isolated fibular fractures, and 60 had tibiofibular fractures. According to the Danis-Weber classification, there were 3 cases of type A fractures, 37 cases of type B fractures, and 31 cases of type C fractures. The average age of the patients was 48.08 ± 14.84 years, with an average follow-up time of 17.41 ± 3.41 months.

All patients treated with the STMIPO technique provided written informed consent to participate in the study, and the study was approved by the Ethics Committee of our hospital (IRB NO.20211010-020).

## Surgical procedures

### In the STMIPO group

Preoperative assessments included anteroposterior and lateral x-ray examinations of the ankle joint, as well as three-dimensional CT scans to determine the type of fracture. Radiologically, the direction of the fracture line was identified, and the distances from the apex of the fibula to the anterior and posterior edges of the fracture line were measured.

**Figure 1:**
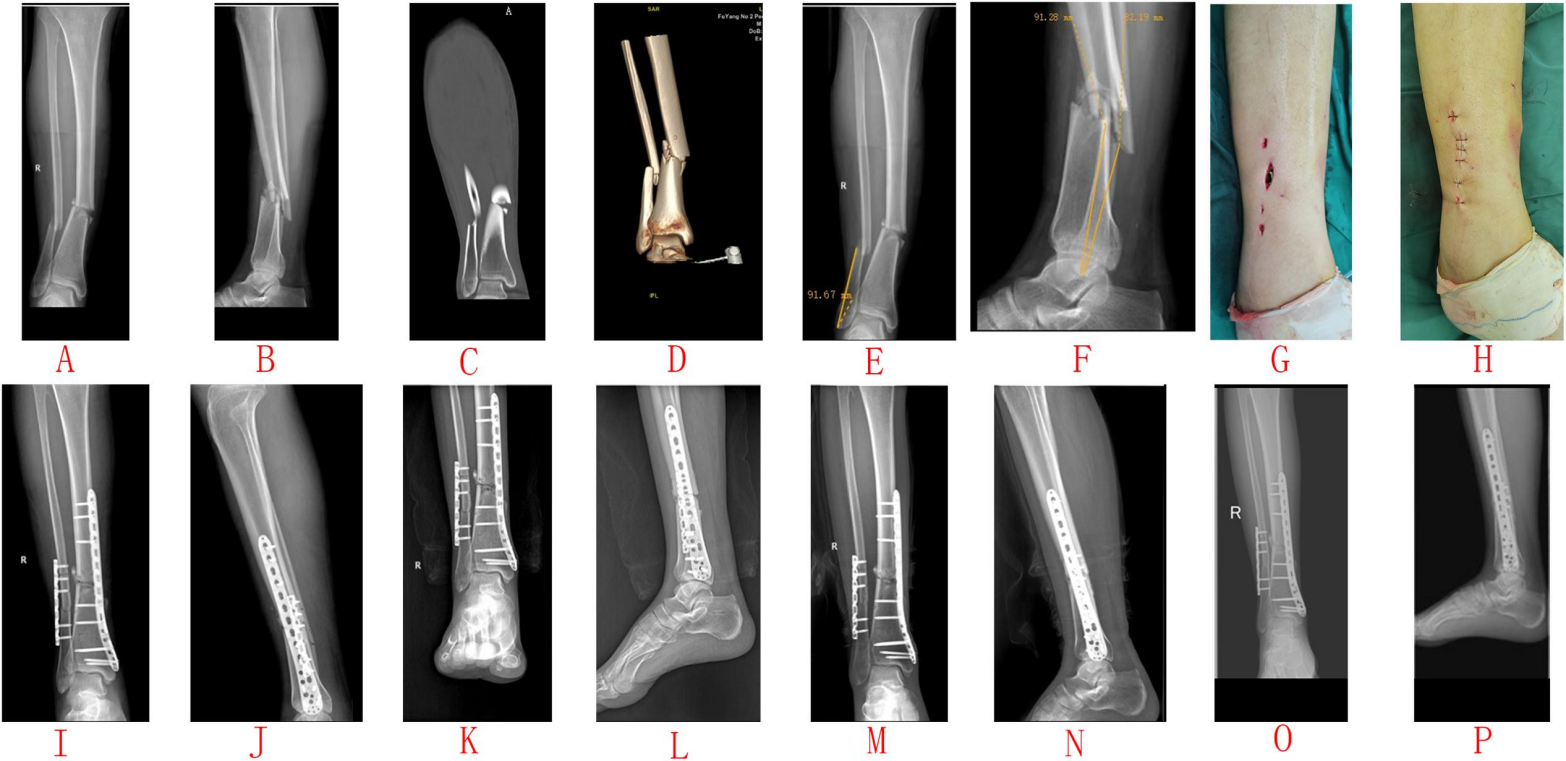
A 51-year-old male with a right tibiofibular fracture (weber type C), treated using the STMIPO technique for the fibula fracture. **(A,B)** Preoperative anteroposterior and lateral x-rays; **(C) (D)** 3D CT scan of the tibia and fibula; **(E,F)** Preoperative measurement of relevant indicators; **(G,H)** Surgical incision; **(I,J)** Postoperative anteroposterior and lateral x-rays; **(K,L)** Anteroposterior and lateral x-rays one month postoperative; **(M,N)** Anteroposterior and lateral x-rays three months postoperative; **(O,P)** Anteroposterior and lateral x-rays six months postoperative.

Epidural or general anesthesia is selected, and the patient is positioned supine with the affected limb elevated at the hip. The use of a tourniquet is decided based on the predicted intraoperative blood loss. Using the lateral malleolus apex as a surface reference point, the incision direction is marked based on preoperative imaging measurements. The incision is made along the fracture direction. But, the incision length was not the full length of the fracture line; rather, it was limited to approximately the width of the selected plate (typically 3–4 cm), sufficient only for plate insertion and limited direct visualization of the fracture ends. Preoperatively, the course of the superficial peroneal nerve and sural nerve was mapped using anatomical landmarks. The incision was intentionally positioned in a nerve-safe zone, typically between the superficial peroneal nerve anteriorly and the sural nerve posteriorly. The skin, subcutaneous tissue, fascia, and three-fourths of the periosteum are incised layer by layer to expose part of the fracture site. Two 3.0 mm diameter Kirschner wires are drilled into the proximal and distal fracture fragments of the fibula, respectively. These wires serve as handles for reduction. The assistant exposes the fracture site, while the main surgeon holds the handles to perform reduction under direct vision. Once satisfactory reduction is achieved, the assistant temporarily fixes the fracture ends with 1.5 mm or 2.0 mm Kirschner wires. Fluoroscopy is used to confirm proper alignment of the fracture ends. The periosteum is then sutured, the two 3.0 mm Kirschner wires are removed, and a suitable length plate is selected. The chosen plate is inserted between the periosteum and subcutaneous tissue along the fibular shaft towards the proximal end. Once the plate is fully inserted, it is moved distally to the appropriate position, and screws are percutaneously inserted one by one. After the surgery, fluoroscopy is used again to observe the reduction and position of the plate (Figure 1).

### In the ORIF group

After successful anesthesia, the patient is placed in a supine position with the affected limb elevated at the hip, and a tourniquet is applied at the base of the thigh. A parallel incision approximately 10 centimeters in length is made on the lateral side of the fibular fracture site to expose the fracture site. Subsequently, the physician reduces the fracture under direct visualization and selects an appropriate length of steel plate to stabilize the fracture fragments in the desired position. The screws are then inserted one by one into the plate to ensure secure fixation. Intraoperative fluoroscopy is also used to assess the reduction of the fracture and the position of the plate to ensure they meet the expected criteria.

## Follow-up and observation indicators

Postoperative follow-ups were conducted regularly at 1, 2, 3, 6, 9, and 12 months after surgery. The following data were recorded: operation time, intraoperative blood loss (combined gravimetric and volumetric method), bone healing time of the affected limb, incision size, America Orthopedic Foot & Ankle Society (AOFAS) ankle function score (15), and complications related to fracture internal fixation (such as bone infection, delayed bone healing, and plate exposure).

AOFAS scores were assessed only in patients with isolated fibular fractures (*n* = 24). For patients with combined tibiofibular fractures, AOFAS scores were not collected because ankle functional recovery in these patients is influenced by multiple tibia-related factors (surgical approach, fixation method, fracture comminution, and syndesmotic injury), making it methodologically invalid to isolate the effect of the fibular surgical technique.

## Statistical methods

Data were analyzed using SPSS 26.0 software. Measurement data were expressed as mean ± standard deviation (SD) and analyzed using the t-test. Count data were analyzed using the chi-square (*χ*^2^) test. A *P*-value of less than 0.05 was considered statistically significant. *post-hoc* power analysis was performed using G*Power 3.1.9.7. For continuous outcomes (incision length, blood loss), the achieved power was calculated based on observed mean differences and pooled standard deviations. For categorical outcomes (complication rate), Fisher's exact test power was computed using the observed proportions. An *α* level of 0.05 was used.

## Results

All patients achieved osseous union of the fibula. Statistical analysis showed that the STMIPO group had advantages over the ORIF group in terms of surgical incision length (3.28 ± 0.50 cm VS 9.96 ± 1.69 cm, *P* = 0.00), intraoperative blood loss (11.08 ± 4.70 mL VS 105.35 ± 27.20 mL, *P* = 0.00), and postoperative complications (4/74 VS 12/71, *P* = 0.03). However, the ORIF group had a shorter operation time to compare with STMIPO group (42.31 ± 9.05 min VS 60.15 ± 11.10 min). There was no significant difference between the two groups in fracture healing time and postoperative ankle AOFAS scores for isolated fibular fractures. In the STMIPO group, 4 cases experienced implant-related pain, which was controlled after implant removal. In the ORIF group, 6 cases had implant-related pain controlled after implant removal, with 1 case experiencing plate exposure and 4 cases of wound infection, all of which healed after implant removal and wound closure (Table 1).

**Table 1:** Basic data of the patients from the STMIPO group and ORIF group.

	STMIPO group (*n* = 74)	ORIF group (*n* = 71)	*P* value
*Baseline characteristics*			
Mean age (year)	47.89 ± 17.26	48.08 ± 14.84	0.94
Gender			0.91
Male	40	39	
Female	34	32	
Danis Weber classification			0.78
Weber A	5	3	
Weber B	39	37	
Weber C	30	31	
Tibial Fracture			0.74
No	13	11	
Yes	61	60	
*Clinical outcomes*			
Length of Incision (cm)	3.28 ± 0.50	9.96 ± 1.69	<0.001
Time of Surgery (minute)^a^	60.15 ± 11.10	42.31 ± 9.05	<0.001
Blood Loss (mL)	11.08 ± 4.70	105.35 ± 27.20	<0.001
Follow-up Time (month)	16.85 ± 1.62	17.41 ± 3.41	0.21
Time of bone union (week)	17.89 ± 1.23	18.08 ± 1.02	0.34
Complication	4 (5.4%)	12 (16.9%)	0.03
Wound infection	0	4	
Bone nonunion	0	1	
Hardware-related pain	4	6	
Plate exposure	0	1	

a The time from fibular incision to incision closure.

To assess whether the treatment effect of STMIPO differed between isolated fibular fractures and combined tibiofibular fractures, we performed a prespecified subgroup analysis stratified by fracture pattern (Supplementary Table 1).

The direction and magnitude of the treatment effect were consistent across both subgroups. In both isolated fractures (*n* = 24) and combined fractures (*n* = 121), STMIPO was associated with significantly shorter incision lengths, less intraoperative blood loss, and shorter operation times compared with ORIF (all *P* < 0.001). No significant differences were observed in time to bone union in either subgroup.

AOFAS scores were only assessed in the isolated fibular fracture subgroup (*n* = 24) and showed no significant difference between STMIPO and ORIF (*P* = 0.16). However, *post-hoc* power analysis indicated that this subgroup was underpowered for this comparison (achieved power: 21%). Therefore, the AOFAS finding should be interpreted with caution.

## Discussion

The name ’Second to MIPO’ was chosen deliberately to reflect the theoretical consideration that STMIPO causes slightly more periosteal injury than pure MIPO. In practice, however, this trade-off appears justified. In the present study, STMIPO achieved anatomical reduction under direct vision while maintaining minimal soft tissue damage, as evidenced by significantly smaller incisions (3.28 cm VS 9.96 cm) and less blood loss (11.08 mL VS 105.35 mL). Importantly, despite the theoretical concern about periosteal injury, bone healing time was comparable between the STMIPO and ORIF groups (17.89 weeks VS 18.08 weeks, *P* = 0.34), with no cases of delayed union or nonunion in the STMIPO group. These findings suggest that the minimal additional periosteal exposure required for direct-vision reduction does not adversely affect fracture healing.

This study represents, to our knowledge, the first large-scale comparative evaluation of STMIPO versus ORIF specifically for distal one-third fibular fractures. While our previous work established the technical feasibility of STMIPO across various limb fractures, the present investigation advances the evidence base from ‘feasibility’ to ‘comparative clinical effectiveness’ by providing quantitative outcome comparisons in a dedicated, high-value fracture scenario. The key findings—significantly smaller incisions (3.28 cm VS 9.96 cm), reduced blood loss (11.08 mL VS 105.35 mL), fewer complications (5.4% VS 16.9%), and comparable functional outcomes—support the clinical utility of STMIPO for this specific fracture type.

The fibula has an anatomical characteristic where the proximal three-fourths are surrounded by muscle, and the distal one-fourth is subcutaneous (16). This makes surgery for distal fibular fractures prone to higher risks of wound non-healing or infection. Relevant studies have shown that the incidence of wound infection or plate exposure after plate and screw fixation of distal fibular fractures is approximately 6% (17, 18). The MIPO technique can reduce postoperative complications for distal fibular fractures; however, it has the drawback of difficult reduction, making it challenging to achieve anatomical reduction. Additionally, it is difficult to implement for Danis-Weber type A fractures (19–22). To overcome these challenges, this study applied the STMIPO technique to treat distal fibular fractures. All patients experienced good wound healing with no skin complications. Furthermore, the STMIPO technique is suitable for all types of fractures.

Classic surgical incision for fibula fractures runs parallel to the direction of the fibula shaft, which facilitates fracture reduction and plate placement (23). However, the main disadvantage of this incision method is the increased risk of damage to surrounding soft tissues. In this study, the authors chose small incisions aligned with the direction of the fracture line. Postoperatively, all patients demonstrated excellent skin wound healing, with no occurrences of wound infection. Additionally, patients expressed high satisfaction with the appearance of the wounds. Based on the anatomical features and vascular distribution of the fibula and surrounding tissues, the authors believe that incisions aligned with the direction of the fracture line can reduce damage to the vascular structures of the fibula and surrounding soft tissues. However, this theory currently lacks sufficient empirical evidence to support it, and further research is needed to validate its effectiveness.

The advantage of open reduction and internal fixation in the treatment of fractures lies in its ability to fully expose the fracture ends, facilitating anatomical reduction of the fibula fracture (4). In this study, the authors employed the STMIPO technique, which not only achieved the goal of anatomical reduction but also met the objectives of minimally invasive surgery. The study demonstrated that the STMIPO technique in the treatment of fibula fractures performed better in terms of intraoperative blood loss, and incision length compared to the traditional ORIF method. The intraoperative convertibility of STMIPO — the ability to readily extend the incision for limited open reduction if needed — serves as a ’safety net’ for the surgeon. Although no conversion was required in the present fibular fracture cohort, this feature has been clinically validated in our practice for other fracture types and distinguishes STMIPO from pure MIPO, which lacks this option. AOFAS scores were assessed only in the isolated fibular fracture subgroup (*n* = 24). In this exploratory analysis, no significant difference was observed between the STMIPO and ORIF groups (97.31 ± 0.95 vs. 96.82 ± 0.75, *P* = 0.16). However, *post-hoc* power analysis showed that this subgroup was severely underpowered (achieved power 21%). Therefore, this finding does not allow us to conclude that the two techniques are equivalent in terms of ankle function. The comparable functional outcomes observed in this small subgroup should be considered hypothesis-generating. The primary conclusion that STMIPO offers advantages in incision length, blood loss, and complications is based on the full cohort analysis (*n* = 145) and is not dependent on the AOFAS finding.

Based on the full cohort analysis (*n* = 145), the authors believe that the STMIPO technique has significant advantages in the treatment of fibular fractures. This technique not only allows for anatomical reduction of the fracture under direct visualization but also reduces the risk of complications. Additionally, the STMIPO technique has achieved good aesthetic results postoperatively and has received high satisfaction from patients. These results indicate that the STMIPO technique is a safe and effective option for the treatment of fibular fractures and is worth promoting and applying in clinical practice.

Several limitations of this study should be acknowledged. First, this was a retrospective study without randomization. Patient allocation was determined by admission date and surgeon schedule, which may introduce selection bias. Although baseline characteristics were similar between groups, unmeasured confounders cannot be excluded. Second, the two techniques were performed by different surgeons, introducing operator bias. Although both surgeons have comparable seniority and case volume, and the ORIF surgeon had a shorter operation time (suggesting at least equal efficiency), we cannot completely exclude the possibility that the observed advantages of STMIPO partly reflect differences in surgical skill rather than the technique itself. Third, tourniquet use was not standardized; it was used selectively in the ORIF group based on predicted blood loss. This may increase outcome variance but does not systematically bias the comparison. Fourth, functional assessment using the AOFAS score was only available for the isolated fibular fracture subgroup (*n* = 24, 16.5% of the cohort). This subgroup was small, which limits the statistical power and generalizability of the findings. *post-hoc* power analysis showed that this subgroup was underpowered to detect a clinically meaningful difference (achieved power: 21%). Therefore, the AOFAS result should be interpreted as exploratory rather than definitive, and the lack of a significant difference does not constitute evidence of functional equivalence. We also acknowledge that the AOFAS score is not applicable to combined tibiofibular fractures, as ankle function in those patients is dominated by tibial healing and fixation. For the 121 patients with combined fractures, functional outcomes were not assessed, which is a significant limitation of this study. Future studies should consider using functional instruments validated for combined fractures or should stratify randomization by fracture pattern to enable separate functional analysis.Fifth, the lack of blinding of outcome assessors could bias subjective measures such as AOFAS scores, although objective outcomes like incision length and blood loss are less susceptible. Sixth, the average follow-up of 17 months, while sufficient for bone healing and early complications, may not capture long-term outcomes such as post-traumatic arthritis or hardware-related pain requiring removal beyond two years. Future prospective randomized controlled trials with larger sample sizes, blinded assessment, and longer follow-up are needed to confirm our findings, and for combined tibiofibular fractures we recommend using functional instruments that isolate ankle-specific function or stratifying randomization by fracture pattern.

## Data Availability

The raw data supporting the conclusions of this article will be made available by the authors, without undue reservation.

## References

[1] Elsoe R Ostgaard SE Larsen P. Population-based epidemiology of 9767 ankle fractures. Foot Ankle Surg. (2018) 24:34–9. 10.1016/j.fas.2016.11.002 29413771

[2] Pflüger P Harder F Müller K Biberthaler P Crönlein M. Evaluation of ankle fracture classification systems in 193 trimalleolar ankle fractures. Eur J Trauma Emerg Surg. (2022) 48:4181–8. 10.1007/s00068-022-01959-2 35348840 PMC9532295

[3] Kang HJ Lee JW Kwon YM Kim SJ. Epidemiology of ankle fractures in Korea: a nationwide population-based study. J Korean Med Sci. (2022) 37:e288. 10.3346/jkms.2022.37.e288 36193640 PMC9530309

[4] Bäcker HC Greisberg JK Vosseller JT. Fibular plate fixation and correlated short-term complications. Foot Ankle Spec. (2020) 13:378–82. 10.1177/1938640019873539 31538819

[5] Vasanad GH Antin SM Akkimaradi RC Policepatil P Naikawadi G. The role of fibular fixation in distal tibial fractures. J Clin Diagn Res. (2016) 10:RC12–14. 10.7860/JCDR/2016/7249.7833 27190908 PMC4866206

[6] Javdan M Tahririan MA Nouri M. The role of fibular fixation in the treatment of combined distal tibia and fibula fracture: a randomized, control trial. Adv Biomed Res. (2017) 6:48. 10.4103/2277-9175.205190 28620592 PMC5433694

[7] Prasad M Yadav S Sud A Arora NC Kumar N Singh S. Assessment of the role of fibular fixation in distal-third tibia-fibula fractures and its significance in decreasing malrotation and malalignment. Injury. (2013) 44:1885–91. 10.1016/j.injury.2013.08.028 24074830

[8] Jung G-H Chung H Baek S-H Sohn H-S. Percutaneous bridge plating of extra-articular distal fibular fracture for the management of distal tibia type III open fracture. Asian J Surg. (2021) 44:363–8. 10.1016/j.asjsur.2020.09.016 33092962

[9] Ahn JH Cho SH Jeong M Kim Y-C. One-Third tubular plate remains a clinically good option in Danis-weber type B distal fibular fracture fixation. Orthop Surg. (2021) 13:2301–9. 10.1111/os.13160 34708569 PMC8654649

[10] Vance DD Swindell HW Greisberg JK Vosseller JT. Outcomes following posterior and posterolateral plating of distal fibula fractures. Foot Ankle Spec. (2019) 12:246–52. 10.1177/1938640018788433 30015505

[11] Moriarity A Ellanti P Mohan K Fhoghlu CN Fenelon C McKenna J. A comparison of complication rates between locking and non-locking plates in distal fibular fractures. Orthop Traumatol Surg Res. (2018) 104:503–6. 10.1016/j.otsr.2018.03.001 29581071

[12] El Fatayri B Bulaïd Y Djebara A-E Havet E Mertl P Dehl M. A comparison of bone union and complication rates between locking and non-locking plates in distal fibular fracture: retrospective study of 106 cases. Injury. (2019) 50:2324–31. 10.1016/j.injury.2019.10.001 31635907

[13] Strauss EJ Alfonso D Kummer FJ Egol KA Tejwani NC. The effect of concurrent fibular fracture on the fixation of distal tibia fractures: a laboratory comparison of intramedullary nails with locked plates. J Orthop Trauma. (2007) 21:172–7. 10.1097/BOT.0b013e3180332dd2 17473753

[14] Li W Zhao Y Liu L Yu H Xie Z Zhuang Q. Limb fractures treated with the novel plate osteosynthesis application technique: second to minimally invasive plates osteosynthesis. J Am Acad Orthop Surg Glob Res Rev. (2024) 8(3):e24.00017. 10.5435/JAAOSGlobal-D-24-00017 38466989 PMC10927324

[15] Dawson J Boller I Doll H Lavis G Sharp R Cooke P. Responsiveness of the Manchester-Oxford foot questionnaire (MOXFQ) compared with AOFAS, SF-36 and EQ-5D assessments following foot or ankle surgery. J Bone Joint Surg Br. (2012) 94:215–21. 10.1302/0301-620X.94B2.27634 22323689

[16] Anetai H Kinose S Sakamoto R Onodera R Kato K Kawasaki Y. Anatomic characterization of the tibial and fibular nutrient arteries in humans. Anat Sci Int. (2021) 96:378–85. 10.1007/s12565-020-00600-9 33453037

[17] Schepers T Van Lieshout EMM De Vries MR Van der Elst M. Increased rates of wound complications with locking plates in distal fibular fractures. Injury. (2011) 42:1125–9. 10.1016/j.injury.2011.01.009 21329921

[18] Poutoglidou F Metaxiotis D Vasiliadis AV Mpeletsiotis A. Plate fixation versus percutaneous Rush pinning for osteosynthesis of the fibula in pilon fractures. A retrospective comparative study. Hippokratia. (2021) 25:63–8. 35937519 PMC9347338

[19] Park YU Kim SJ Kim HN. Minimally invasive plate osteosynthesis using the oblong hole of a locking plate for comminuted distal fibular fractures. J Orthop Surg Res. (2021) 16:281. 10.1186/s13018-021-02441-2 33906661 PMC8077965

[20] Unlu S Catma MF Bilgetekin YG Altay M Ates Y Bozkurt M. Minimally invasive plate osteosynthesis of distal tibia and fibular fractures through a single distal anterolateral incision. J Foot Ankle Surg. (2015) 54:1081–4. 10.1053/j.jfas.2015.06.009 26190782

[21] Iacobellis C Chemello C Zornetta A Aldegheri R. Minimally invasive plate osteosynthesis in type B fibular fractures versus open surgery. Musculoskelet Surg. (2013) 97:229–35. 10.1007/s12306-013-0292-x 23900920

[22] Krenk DE Molinero KG Mascarenhas L Muffly MT Altman GT. Results of minimally invasive distal fibular plate osteosynthesis. J Trauma. (2009) 66:570–5. 10.1097/TA.0b013e31818936ff 19204536

[23] Badenhorst D Terblanche I Ferreria N Burger MC. Intramedullary fixation versus anatomically contoured plating of unstable ankle fractures: a randomized control trial. Int Orthop. (2020) 44:561–8. 10.1007/s00264-020-04482-4 31980861

