# Supplementary material for: Clinical efficacy of STMIPO in the management of distal one-third fibular fractures
Source: Front Surg. 2026 Jul 6;13:1848647. doi: 10.3389/fsurg.2026.1848647 (PMC13381843; doi:10.3389/fsurg.2026.1848647)
Supplement: Supplementary file 1 [file Table1.docx]

| Outcome | Isolated fibular fractures | | | Combined tibiofibular fractures | | |
| --- | --- | --- | --- | --- | --- | --- |
|  | STMIPO Group (n=13) | ORIF Group (11) | P value | STMIPO Group (n=61) | ORIF Group (60) | P value |
| Incision length (cm) | 3.35±0.52 | 9.55±1.44 | <0.001 | 3.27±0.50 | 10.03±1.73 | <0.001 |
| Blood loss (mL) | 10.00±3.54 | 89.09±28.44 | <0.001 | 11.31±4.91 | 108.33±26.11 | <0.001 |
| Time of Surgery（minute）^a^ | 61.38±9.40 | 42.55±7.08 | <0.001 | 59.89±11.48 | 42.27±9.41 | <0.001 |
| Time of bone union （week） | 18.00±1.63 | 18.45±1.44 | 0.48 | 17.87±1.15 | 18.02±1.16 | 0.48 |
| AOFAS score^b^ | 97.31±0.95 | 96.82±0.75 | 0.16 |  |  |  |

**Supplementary Table 1.Subgroup analysis of clinical outcomes stratified by fracture pattern (isolated fibular fractures vs. combined tibiofibular fractures)**

Note:^a^ The time from fibular incision to incision closure.

^b^ AOFAS score was assessed only in patients with isolated fibular fractures.
